# Characterization of a complex chromosomal rearrangement using chromosome, FISH, and microarray assays in a girl with multiple congenital abnormalities and developmental delay

**DOI:** 10.1186/1755-8166-7-50

**Published:** 2014-08-29

**Authors:** Morteza Hemmat, Xiaojing Yang, Patricia Chan, Robert A McGough, Leslie Ross, Loretta W Mahon, Arturo L Anguiano, Wang T Boris, Mohamed M Elnaggar, Jia-Chi J Wang, Charles M Strom, Fatih Z Boyar

**Affiliations:** 1 Cytogenetics Department, Quest Diagnostics Nichols Institute, 33608 Ortega Hwy, San Juan Capistrano, California 92675, USA; 2 Quest Diagnostics, 695 South Broadway, Denver, Colorado 80209, USA; 3 Quest Diagnostics, 8401 Fallbrook Avenue , West, Hills, California 91304, USA

**Keywords:** Complex chromosomal rearrangement (CCR), microarray, CMA, Whole chromosome painting (WCP) FISH

## Abstract

Complex chromosomal rearrangements (CCRs) are balanced or unbalanced structural rearrangements involving three or more cytogenetic breakpoints on two or more chromosomal pairs. The phenotypic anomalies in such cases are attributed to gene disruption, superimposed cryptic imbalances in the genome, and/or position effects. We report a 14-year-old girl who presented with multiple congenital anomalies and developmental delay. Chromosome and FISH analysis indicated a highly complex chromosomal rearrangement involving three chromosomes (3, 7 and 12), seven breakpoints as a result of one inversion, two insertions, and two translocations forming three derivative chromosomes. Additionally, chromosomal microarray study (CMA) revealed two submicroscopic deletions at 3p12.3 (467 kb) and 12q13.12 (442 kb). We postulate that microdeletion within the ROBO1 gene at 3p12.3 may have played a role in the patient’s developmental delay, since it has potential activity-dependent role in neurons. Additionally, factors other than genomic deletions such as loss of function or position effects may also contribute to the abnormal phenotype in our patient.

## Background

Complex chromosomal rearrangements (CCRs) are balanced or unbalanced structural rearrangements involving three or more cytogenetic breakpoints on two or more chromosomes [1-5]. The apparently balanced CCRs range from simple three-way exchanges between three chromosomes to highly complex translocations involving many chromosomes and multiple breaks [6].

Chromosomal rearrangements may occur via several mechanisms [7], including non-allelic homologous recombination (NAHR) [8,9] and nonhomologous end-joining (NHEJ), which both lead to deleted or duplicated genomic segments. However, a number of disease-associated rearrangements are not explained readily by either the NAHR or simple NHEJ recombination mechanisms. Fork stalling and template switching (FoSTeS) and microhomology-mediated break-induced replication (MMBIR) have been described as a mechanism associated with complex rearrangements caused by abnormal DNA replication [7,10,11]. More recently, Liber et al. and Tsai et al. proposed a mechanism in which simultaneous double-strand DNA breaks were induced by an unknown stimulus, such as free radicals or ionizing radiation. This is followed by joining of the break fragments in the wrong place due to the microhomology shared by these regions [12,13].

Balanced and unbalanced CCRs are associated with a significant risk of mental retardation and phenotypic anomalies attributable to gene disruption, cryptic imbalances and/or from position effects [14-18]. Fluorescence in situ hybridization (FISH) and/or high resolution chromosomal microarray studies have identified cryptic CCRs as a cause of abnormal phenotype in a significant number of patients with apparently balanced chromosomal rearrangements [19-23].

We report a patient with multiple congenital anomalies and developmental delay who presented with a CCR involving three chromosomes 3, 7 and 12. G-banding, chromosomal microarray (CMA), and FISH were performed to clarify the nature of this complex abnormality.

## Case presentation

### Case report

The patient was a 14-year-old female who presented clinically with developmental delay and multiple congenital anomalies including abnormal teeth and abnormal faces. No further clinical information was available regarding this patient.

## Methods and results

**Figure 1:**
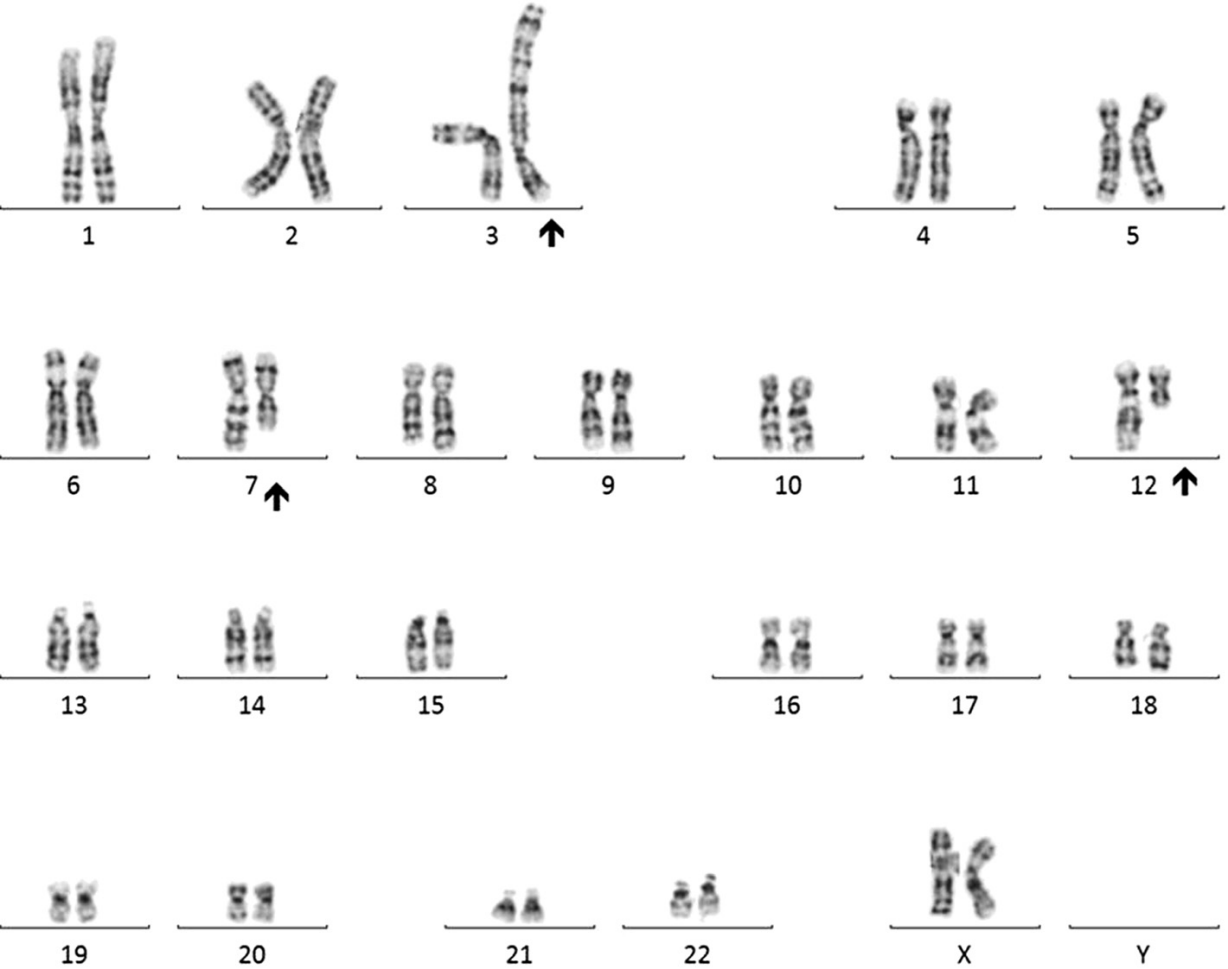
**A karyotype showing a complex rearrangement resulting in derivative chromosomes 3,7,and 12.**

**Figure 2:**
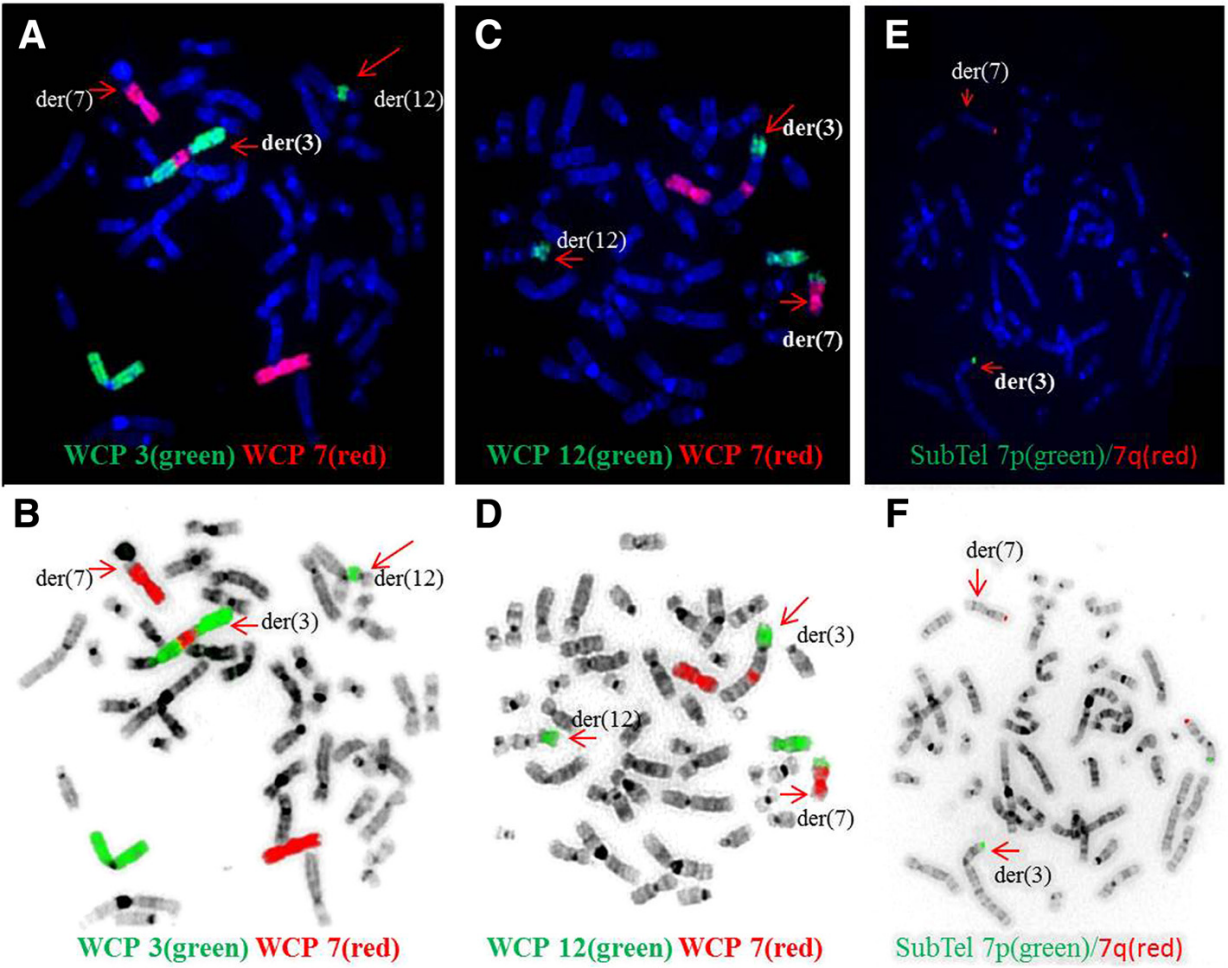
**FISH results with whole**-**chromosome painting** (**WCP**) **for chromosomes 3 and 7 (A/B) and 7 and 12 (C/D), and subtelomeric FISH for chromosome 7(E/F).** The bottom row of images are the same FISH images shown in the top row but with inverted DAPI stain. The chromosomes involved in the CCR are depicted by red arrows. A/B: WCP3 (green) paints the normal chromosome 3 part of the der (3) and der (12); WCP7 (red) paints the normal chromosome 7, part of der (7), and the inserted part in der (3). C/D: WCP12 (green) paints the normal chromosome 12, part of der (12), translocated part to der (3), and translocated part to der (7); WCP7 (red) paints the normal chromosome 7 and part of der (7) and inserted part to der (3). E/F: Subtelomeric 7p (green) and 7q (red) are shown in the normal chromosome 7. The subtelomeric 7p probe of the other chromosome 7 is located on der (3) after translocation to chromosome 12.

**Figure 3:**
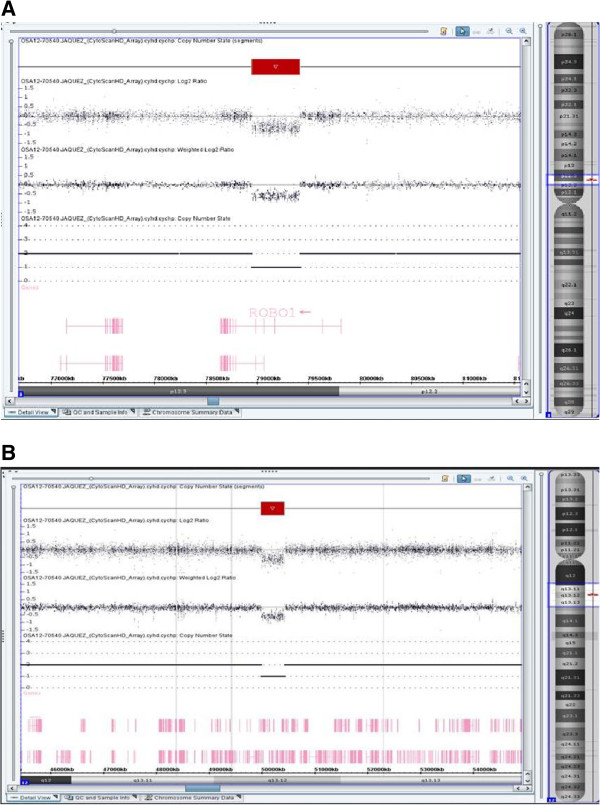
**SNP-array results for chromosome 3 (A) and chromosome 12 nB).** The log2 ratio, weighted log2 ratio, and copy number state indicate the deleted regions for both chromosomes 3 and 12 and one segment of loss of heterozygosity for chromosome 12.

Peripheral blood sample from the patient was referred to our laboratory for chromosome analysis. Metaphase chromosomes were prepared according to standard procedures [24-27]. Analysis of the GTG-banded metaphase chromosomes at the resolution level of 400 bands revealed highly complex rearrangements including one inversion, two insertions, and two translocations involving seven breakpoints at chromosomes 3, 7, and 12 (Figure 1). All of the rearrangements were confirmed by FISH studies using whole-chromosome painting (WCP) probes for chromosomes 3, 7, and 12 (Abbott Molecular, VYSIS, Chicago, IL and Cytocell, Cambridge, UK) and subtelomere 7p/7q probes (Abbott Molecular, VYSIS) (Weise et al. 2008) (Figure 2). WCP FISH results confirmed the ins (3;7), the t (3;12), and the cryptic der (7) t (7;12). The subtelomere 7p probe present on der (3) qter further characterized the translocation between chromosome 7 and chromosome 12. This portion of 7p (7pter) had been translocated to chromosome 12 before being translocated jointly with a segment of chromosome 12 to chromosome 3. This type of CCR has been classified as type III [6], since the number of breaks was greater than the number of affected chromosomes and it included one insertion. SNP-microarray study was performed in order to rule out cryptic copy number variations. Genomic DNA was extracted from whole blood using the Gentra Puregene kit (Qiagen-Sciences, Maryland, USA). Microdeletion/microduplication screening was performed for the proband and his mother, and available half-brothers using an SNP-array platform (CytoScan HD; Affymetrix, Santa Clara, CA), following the manufacturer’s instructions. The CytoScan HD array has 2.67 million probes, including 1.9 million copy number probes and 0.75 million SNP probes. Array data were analyzed using the Chromosome Analysis Suite (ChAS) (Affymetrix, Inc.) software v 2.0. CMA testing revealed two genomic segments in the proband consistent with deletions of approximately 467 kb at 3p12.3 and 442 kb at 12q13.12 (Figure 3A, B). Although, we could not confirm, the deletion at 3p12.3 is likely to encompass the breakpoint on the derivative chromosome 3, where the insertion of chromosome 7 material occurred. This microdeletion encompassed two exons of ROBO1 gene, extended from 78,952,028 to 79,418,897 bp (UCSC genome Browser; http://genome.ucsc.edu/; hg19 release). Similarly, the deletion at 12q13.12 is likely resulted from the translocation between chromosomes 3 and 12. This microdeletion extended from 49,988,357 to 50,429,906 bp and encompassed thirteen genes. CMA testing of the mother detected no dosage abnormality (gain or loss). The father was not available for follow up.

The combination of cytogenetic, FISH, and array analysis revealed a complex rearrangement with nine breakpoints:

46,××, der (3) (3pter → 3p12::7q11.2 → 7q22::3q27 → 3p12::12q13 → 12q24.3::7p22 → 7pter), der (7) (12qter → 2q24.3::7p22 → 7q11.2::7q22 → 7qter), der (12) (12pter → 12q13::3q27 → 3qter).arr [hg19] 3p12.3(78,952,028-79,418,897) ×1, 12q13.12 (49,988,357-50,429,906)×1.

## Discussion

The disease-associated CCRs are frequently used to establish the genotype-phenotype relationship [28,29]. A combination of several different approaches, including karyotype, FISH, and CMA studies, has been useful in identifying several disease-associated genes and regions [28-35]. The phenotype of our patient with multiple congenital abnormalities and developmental delay with the apparently balanced CCR led us to perform CMA testing to rule out cryptic copy number variations (CNVs). According to the NCBI Map Viewer (http://www.ncbi.nlm.nih.gov/mapview/), the 3p12.3 deletion was within the *ROBO1* gene, while the deletion at 12q13.12 involved thirteen genes (FAM186B, PRPF40B, FMNL3, TMBIM6, NCKAP5L, BCDIN3D-AS1, BCDIN3D, FAIM2, LOC283332, AQP2, AQP5, AQP6, RACGAP1). It is not clear if these copy number losses are de novo or paternally inherited, since the patient’s father was not available for follow up studies. The *ROBO1* gene encodes a receptor that is a member of the neural cell adhesion molecule (NCAM; 116930) family of receptors, acting as an axon guidance receptor. *ROBO1* may play a role in neuronal development, and its disruption may predispose humans to developmental dyslexia [36,37]. Among the genes deleted at 12q13 three are OMIM genes including *NCKAP5L* (OMIM 615104), *AQP2* (OMIM 107777) and *AQP5* (OMIM 600442). *NCKAP5L* gene encodes a protein involved in proteolysis, GTPase-mediated signaling, cytoskeletal organization, and other pathways. Furthermore, neuronal depolarization regulates the transcription of these genes, suggesting potential activity-dependent roles in neurons [38]. Mutation in AQP2 is associated with diabetes insipidus and in AQP5 with palmoplantar keratoderma, Bothnian type. However, copy number losses or gains at these loci have not yet been associated with a clinical phenotype. We propose that microdeletion within ROBO1 may play a role in our patient’s developmental delay.

Since the exact genomic location of at least five out of seven breakpoints in our patient is unknown, we can only speculate as to the disruption of genes resulting in loss of function [21,39] or position effects [40] in these chromosomal breakpoints. For example, inversion of 3p12q27 may interrupt the *DYX5* gene (OMIM 606896) at 3p12, which is associated with neurofunctional disorder, developmental dyslexia [41], or speech sound disorder [42]. Furthermore, interruption in *MASP1* (OMIM 257920) located at the distal end (3q27) of the inversion 3 may have resulted in 3MC syndrome, which encompasses four rare autosomal recessive disorders previously designated as the Carnevale, Mingarelli, Malpuech, and Michels syndromes, respectively. The main features of these syndromes are facial dysmorphism, cleft lip and palate, postnatal growth deficiency, cognitive impairment, and craniosynostosis [43]. The other gene interruption may have occurred in the complex der (3) ins (3;7), where one of the breakpoints maps to 7q11.21. This was previously suggested as a candidate region for ectrodactyly, ectodermal dysplasia, and cleft lip/palate syndrome (designated EEC1) [44]. Alternatively, fusion of these genes at the breakpoints where insertion has occurred may generate a gain-of-function mutation [45]. Additional molecular studies are needed in order to determine whether any interruption or disruption of the genes caused by the chromosomal rearrangements.

## Conclusion

Two sub-microscopic deletions resulted from this apparently balanced CCR. Microdeletion within the *ROBO1* gene with potential activity-dependent roles in neurons may have played a role in our patient’s developmental delay. Furthermore, gene disruptions or position effects altering gene regulation by chromosomal rearrangements due to interference with some gene regulatory elements, may have also contributed to our patient’s abnormal phenotype.

## Consent

These studies were performed on anonymized samples received in the clinical laboratory and thus were exempted from the requirement for consent by an opinion for the Western Institutional review Board.

## Abbreviations

CCR: Complex chromosomal rearrangement; SNP-microarray: Single nucleotide polymorphism.

## Competing interests

The authors declare that they have no competing interests.

## Authors’ contributions

MH, First authors; performed analysis, interpretation of the results, drafting and finalizing the manuscript. XY, participated in writing the FISH results. PC, RM and LR Performed the analysis and literature review. FZB reviewed the manuscript. All authors read and approved the final manuscript.
